# Long-term oxygen therapy in precapillary pulmonary hypertension – SOPHA study

**DOI:** 10.1038/s41598-024-70650-w

**Published:** 2024-09-26

**Authors:** Nicola Benjamin, Ishan Echampati, Satenik Harutyunova, Christina Alessandra Eichstaedt, Benjamin Egenlauf, Silvia Ulrich, Ekkehard Grünig, Panagiota Xanthouli

**Affiliations:** 1 Centre for Pulmonary Hypertension, Thoraxklinik Heidelberg gGmbH at Heidelberg University Hospital, Röntgenstraße 1, 69126 Heidelberg, Germany; 2 Translational Lung Research Centre Heidelberg (TLRC), German Centre for Lung Research (DZL), Heidelberg, Germany; 3 Department of Pneumology and Critical Care Medicine, Thoraxklinik Heidelberg gGmbH at Heidelberg University Hospital, Heidelberg, Germany; 4 Pulmonary Clinic, University and University Hospital of Zurich, Zurich, Switzerland; 5 Medical Clinic V, Haematology, Oncology, Rheumatology, University Hospital Heidelberg, Heidelberg, Germany

**Keywords:** Oxygen, Pulmonary hypertension, 6-minutes walking distance, Exercise hypoxemia, Cardiovascular diseases, Respiratory tract diseases

## Abstract

Current guidelines recommend oxygen (O_2_) supplementation in patients with pulmonary hypertension (PH), despite scarce data on long-term O_2_ therapy (LTOT). The aim of this prospective, randomized, controlled trial was to investigate the effect of LTOT in patients with precapillary PH on exercise capacity, clinical parameters and hemodynamics. Patients with precapillary PH under stable therapy and O_2_ desaturations at rest and/or during exercise were randomized to receive LTOT (≥ 16 h/day) or no O_2_ (control group) for 12 weeks. The control group was offered LTOT after 12 weeks. The primary endpoint changes of 6-minute walking distance (6MWD) from baseline to 12 weeks was hierarchically tested: (1) pre-post primary and secondary intervention (2) intervention vs. control group. Secondary endpoints included changes in clinical parameters. Twenty patients were randomized (women n = 14, age 67 ± 11.4 years, mean pulmonary arterial pressure 39.7 ± 12.5 mmHg, 70% functional class III). 6MWD significantly improved by 42.2 ± 34.20 m (*p* = 0.003) within 12 weeks LTOT. The intervention group significantly improved in 6MWD (38.9 ± 33.87 m) compared to the control group (− 12.3 ± 21.83 m, *p* = 0.015). No consistent between-group differences in other parameters were found. LTOT was well tolerated and led to significant improvement of 6MWD. The effect of LTOT should be investigated in larger controlled-trials.

## Introduction

Precapillary pulmonary hypertension (PH) defined as pulmonary arterial hypertension (PAH) and chronic thrombo-embolic pulmonary hypertension (CTEPH) is a rare, chronic, devastating disease associated with pulmonary vasoconstriction leading to right heart failure^1^. PAH and CTEPH patients develop exercise intolerance and dyspnea already at early stages of the disease. Five different classes of medication are approved as treatment options in PAH, two classes in CTEPH, leading to an improvement of hemodynamics, survival and exercise capacity. Short term oxygen (O_2_) administration during right heart catheterization (RHC) has shown to improve mean pulmonary arterial pressure (mPAP) and pulmonary vascular resistance (PVR)^2,3^. O_2_ serves as a vasodilatory, effective, supportive agent rarely having systemic effects.

Hypoxemia often occurs in PAH patients mostly due to ventilation/perfusion defects, right to left shunts, low diffusion capacity and low cardiac output. In the European guidelines on diagnosis and treatment of PH, optimization of supportive therapy, in particular O_2_ supplementation, was supported with a level I class C recommendation, due to the lack of robust data advocating its substitution^1^. Weitzenblum et al. have shown that O_2_ reduced the development of PH in patients with chronic obstructive pulmonary disease^4^. However, O_2_ supplementation did not improve survival among PAH patients with Eisenmenger’s syndrome^5^. On the other hand, recent studies support O_2_ supplementation in PAH patients^6–8^. A short-term O_2_ supply during cardiopulmonary exercise testing (CPET), led to improvement of exercise endurance^6^. Furthermore, the nocturnal O_2_ substitution over a week resulted in an improvement in quality of life (QoL) and 6-minute walking distance (6MWD) among PAH patients^7^, while domiciliary O_2_ substitution over 5 weeks caused an amelioration of 6MWD, World Health Organization (WHO) functional class (FC) and QoL among patients with PAH and CTEPH with desaturations during exercise^8^.

It is already known that patients with COPD show skeletal muscle dysfunction with decreased muscle strength and endurance, increased fatigability, decreased type I and increased amount of type II fibers^9^. Chronic hypoxia leads to an increase in oxidative stress and muscle fiber diameter^10^, with greater oxidative stress and reduction of quadriceps strength in hypoxemic patients, leading to poorer performance^11^. Chronic exercise induces adaptive changes of the musculature including an increase of muscle circulation and improvement of oxygen diffusing capacity. Remodeling of arteries and veins, angiogenesis and functional adaptations lead to an increased blood flow^12^. Whether long-term oxygen supply may help to overcome hypoxia induced skeletal muscle dysfunction and enhance the physiologic response to exercise remains to be investigated.

It remains still unclear, whether long-term O_2_ therapy (LTOT) would translate into improvements of 6MWD, QoL, hemodynamics and disease progression in PAH or CTEPH.

Thus, the aim of this study was to assess the effect of LTOT i.e. O_2_ supplementation ≥ 16 h/day vs. no O_2_ (control group) on 6MWD in patients with PAH and CTEPH (recurrent or persistent after pulmonary endarterectomy) and mild hypoxia at rest and during exercise. Further clinical and echocardiographic parameters, hemodynamics and QoL were also assessed.

## Methods

### Study population and design

This was a randomized, controlled, parallel-group, open-label, monocentric, phase II, clinical trial (SOPHA) with a partial cross-over design to investigate the effect of LTOT (O_2_ group, primary intervention group), ≥ 16 h per day via nasal cannula from liquid O_2_ sources versus no O_2_ (control group) over 12 weeks at the PH Centre in the Thoraxklinik Heidelberg gGmbH at Heidelberg University Hospital in Germany. Adult patients with precapillary PH (i.e. PAH or CTEPH (recurrent or persistent after pulmonary endarterectomy) according to the ERS/ESC PH Guidelines^13^) having mild hypoxia at rest and during exercise (peripheral O_2_ saturation (SpO_2_) ≤ 90%, or partial O_2_ pressure (pO_2_) < 60 mmHg at rest and/or during exercise), who were stable on optimized, targeted PH therapy for at least 6 weeks were assigned to participate in the trial. Randomisation was performed by permuted block randomisation with sealed envelopes. Individuals were excluded, if they had severe desaturations (SpO_2_ < 80% repeatedly or persistent SpO_2_ < 75% during exercise), PH due to left heart- or lung disease, pulmonary venous hypertension severe limitations in pulmonary function test (PFT) with forced expiratory volume in the first second (FEV_1_) < 60% predicted, total lung capacity (TLC) < 60% predicted and significant (> 20%) morphological signs of pulmonary disease in computed tomography of the lungs, inability to follow the protocol as well as unstable conditions requiring pharmacological or other treatment. Current smokers, pregnant individuals or women of child bearing potential without reliable form of birth control were also excluded. Oxygen flow was be determined by titration, aiming at oxygen saturation values of > 90% at rest and during exercise. Further details from the protocol are available under www.clinicaltrials.gov (NCT04207593).

**Fig. 1:**
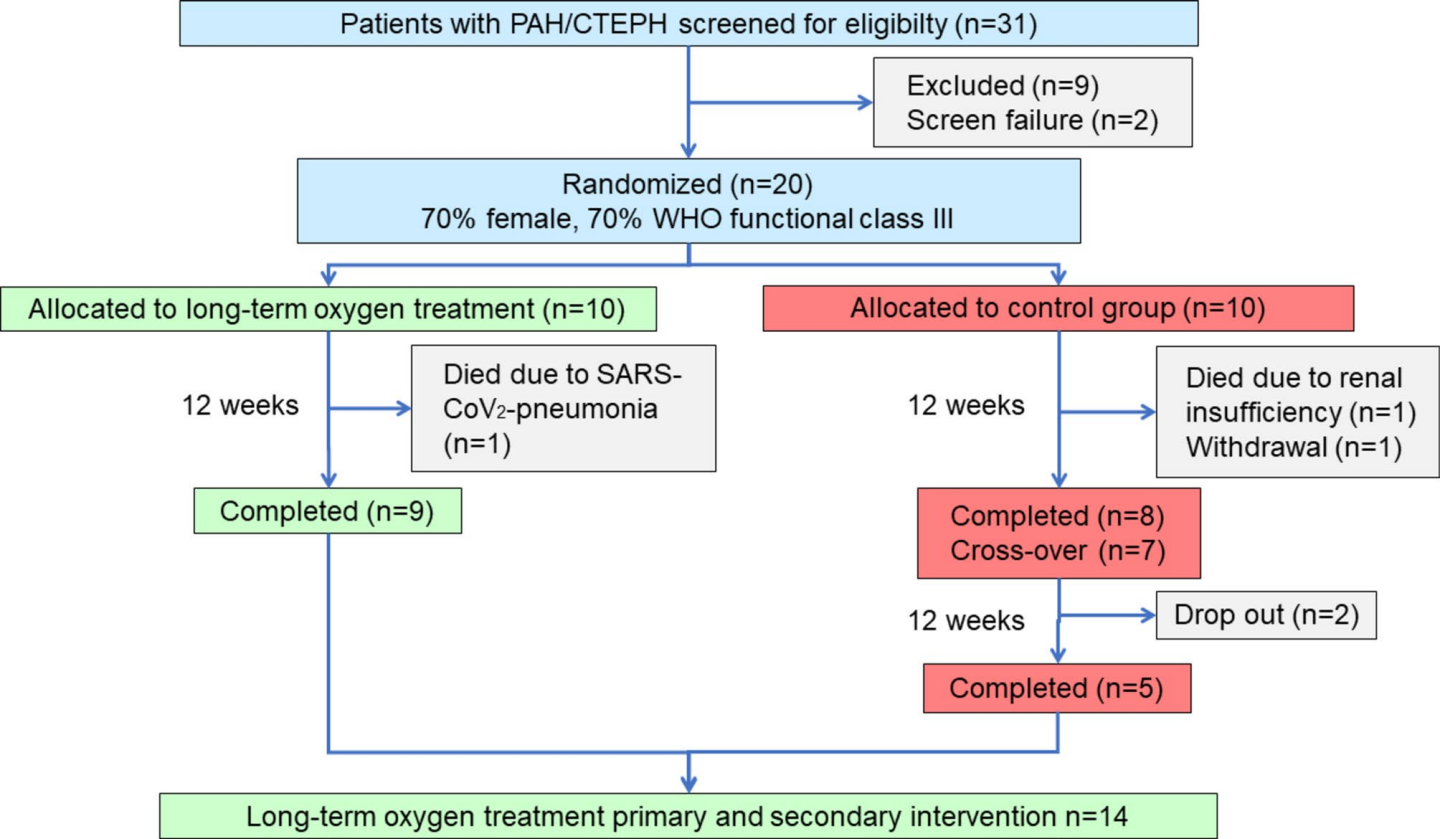
Study flow-chart. The graph provides information on patient-flow, dropouts and group assignment.

After completion of the 12-week study period, patients from the control group were offered to participate in the secondary intervention group with LTOT. Randomization was performed in a 1:1 ratio by block randomization with concealed allocation. Blinding of patients and study physicians was not possible due to the nature of intervention with O_2_. The primary endpoint, 6MWD, was however assessed with and without O_2_ supplementation, to blind the investigators from group assignment. The result from 6MWD without O_2_ supplementation was used to assess effect of LTOT, to exclude short-term effects of O_2_ during the test. Patients documented the intake of O_2_ every day during the study and measured SpO_2_ at home. Participants were contacted by investigators (interim phone visits) at week 4 and week 8 after randomization to assess adherence, clinical status, tolerance and adverse events. Patients randomised in the control group willing to participate in the secondary intervention of the trial were contacted at week 16 and 20 during LTOT treatment, respectively. All patients were called one month after termination of trial participation (Fig. 1). Patients in the control group developing *severe hypoxemia* defined as resting desaturations SpO_2_ ≤ 85% or < 80% over ≥ 10 min during exercise were offered to cross over into the O_2_ arm instantly.

### Efficacy variables

The efficacy of LTOT vs. no O_2_ was assessed at baseline and after 12 weeks of treatment. The primary endpoint was to assess the change in 6MWD over 12 weeks of intervention with LTOT (primary and secondary intervention) and in the primary intervention vs. control group. Medical history, clinical worsening events, clinical examination, WHO FC, electrocardiogram, blood gas analysis from capillarized arterial blood (BGA), PFT including plethysmography and assessment of diffusion capacity for carbon monoxide (DLCO), QoL questionnaire SF-36, 6MWD, echocardiography and laboratory tests including N-terminal natriuretic peptide (NTproBNP) as well as invasive assessment of hemodynamics via RHC were performed. Echocardiographic assessments included right ventricular (RV) and right atrial (RA) area, systolic pulmonary arterial pressure (sPAP), tricuspid annular plane systolic excursion (TAPSE), left-ventricular eccentricity index (LV-EI) and RV-pump function. Invasive hemodynamic measurements via RHC included the assessment of pulmonary arterial pressure (diastolic, systolic and mean: dPAP, sPAP, mPAP), pulmonary arterial wedge pressure (PAWP), PVR, RA pressure (RAP), central venous saturation (SvO_2_), cardiac output (CO) and cardiac index (CI). All parameters were assessed according to current standards in line with the guidelines^13^. RHC was performed without O_2_ substitution. Patients participating in the secondary intervention group underwent these assessments again 12 weeks after the initiation of O_2_. Efficacy parameters were compared intra-individually and secondary between groups.

### Statistical methods

The primary endpoint was analysed in a hierarchical testing strategy with intra- individual analysis of the 6MWD in the primary and secondary intervention group in the intention-to-treat analysis set. Subsequently, the treatment effect was compared between LTOT and the control group, if the hypothesis of the intra-individual analysis was rejected. An increase in 6MWD of at least 35 ± 50 m was expected as minimal important difference based on previous study assumptions^8^. A sample size of at least 8 patients in each group by a dropout rate of 10% led to a final sample size of 18 (9 in each group) to achieve a power of 80%. Due to the distribution of the data, the primary endpoint was analyzed by Mann–Whitney-U-Test instead of the planned ANCOVA with baseline values as covariates. Data are described as mean ± standard deviation (SD) with 95% confidence interval of the mean. The primary analysis set was the intention-to-treat analysis set. As sensitivity analysis set, the data was analyzed with the per protocol analysis set for the primary endpoint. All analyses have been performed using IBM SPSS 27 (SPSS Statistics V.27, IBM Corporation, Somers, New York, USA).

### Study approval and amendments

The protocol and its amendments were approved by the data and safety monitoring and local Ethics Committee of the Medical Faculty of Heidelberg University (Internal number AFmo-839/2018) and Federal Institute for Pharmaceuticals and Medical Products (BfArM) (EudraCT 2018-001387-39) and registered at www.clinicaltrials.gov (NCT04207593) on 23/12/2019. All data were pseudonymized. The study complied with the Declaration of Helsinki in its current version. Written informed consent was provided by all participating patients after detailed information and discussion with the study physician. The study was conducted from January 2019 till January 2023. The study duration was prolonged due to recruitment difficulties and recruitment stop during the SARS-CoV_2_ pandemic. In the initial protocol, 40 patients were estimated to participate, 20 in each group and the assessments included also the performance of a CPET and stress-echocardiography. Due to the SARS-CoV_2_ pandemic the protocol was amended and CPET, stress-echocardiography were not assessed and were removed due to increased infection risks caused by enhanced ventilation.

### Ethical approval

All data were pseudonymised. The protocol and its amendments were approved by the data and safety monitoring and local Ethics Committee of the Medical Faculty of Heidelberg University (Internal number AFmo-839/2018) and Federal Institute for Pharmaceuticals and Medical Products (BfArM) (EudraCT 2018-001387-39) and registered at www.clinicaltrials.gov (NCT04207593) on 23/12/2019. The study complies with the Declaration of Helsinki in its current version. The written informed consent form was signed from the patients upfront the participation in the trial.

## Results

### Included patients and baseline characteristics (Tables 1 and 2)

**Table 1:** Characteristics of the study cohort.

		Whole cohort (n = 20)			Oxygen treatment (n = 10)			Control group (n = 10)		
		Mean ± SD or n and (%)	95% Confidence interval	n*	Mean ± SD or n and (%)	95% Confidence interval	n*	Mean ± SD or n and (%)	95% Confidence interval	n*
Characteristics										
Female sex, no (%)		13 ± 65%			7 ± 70%			6 ± 60%		
Age (years)		66.8 ± 11.4	61.5–72.1	20	67.4 ± 11.4	59.2–75.6		66.2 ± 12.0	57.6–74.8	
Height (cm)		168.3 ± 7.0	165.0–171.6	20	168.1 ± 6.7	163.3–172.9		168.5 ± 7.7	163.0–174.0	
Weight (kg)		83.8 ± 17.7	75.5–92.1	20	86.3 ± 19.7	72.2–100.4		81.3 ± 16.1	69.8–92.8	
Systolic blood pressure (mmHg)		124.3 ± 19.1	115.3–133.2	20	119.0 ± 16.8	107.0–131.0		129.5 ± 20.6	114.8–144.2	
Diastolic blood pressure (mmHg)		74 ± 9.4	69.6–78.4	20	73.0 ± 10.6	65.4–80.6		75.0 ± 8.5	68.9–81.1	
Heart rate at rest (/min)		79.9 ± 13.0	73.8–86.0	20	80.6 ± 12.5	71.6–89.6		79.2 ± 14.2	69.1–89.3	
Type of pulmonary hypertension										
Idiopathic pulmonary arterial hypertension		12 ± 60%			5 ± 50%			4 ± 40%		
Chronic thromboembolic pulmonary hypertension		3 ± 15%			1 ± 10%			2 ± 20%		
Other pulmonary arterial hypertension		5 ± 25%			4 ± 40%			4 ± 40%		
WHO FC, no (%)										
I		2 ± 10%			1 ± 10%			1 ± 10%		
II		5 ± 25%			3 ± 30%			2 ± 20%		
III		13 ± 65%			6 ± 60%			7 ± 70%		
Pulmonary hypertension treatment										
Phosphodiesterase-5-inhibitor		11 ± 55%								
Endothelin receptor antagonist		12 ± 60%								
Soluble guanylate cyclase stimulator		5 ± 25%								
Prostanoids		1 ± 5%								
Calcium channel blockers		5 ± 25%								
Combination therapy										
Double combination regimen		11 ± 55%								
Triple combination regimen		2 ± 10%								
Lung function										
Vital capacity max (l)		2.9 ± 0.7	2.5–3.2	19	2.7 ± 0.7	2.2–3.3	10	3–0 ± 0.7	2.5–3.5	10
Forced expiratory volume in one second (l)		2.1 ± 0.5	1.9–2.4	19	2.1 ± 0.6	1.6–2.5	10	2.2 ± 0.4	1.9–2.6	10
Total lung capacity (l)		5.4 ± 1.2	4.8–6.0	19	5.1 ± 1.2	4.2–5.9	10	5.8 ± 1.2	4.9–6.7	10
Diffusion capacity of the lung for carbon monoxide SB (mmol/min/kPa)		5.7 ± 6.3	2.7–8.8	19	4.0 ± 1.5	3.0–5.1	10	7.6 ± 8.9	0.8–14.5	10
Diffusion capacity of the lung for carbon monoxide/VA (%)		52.7 ± 15.0	45.5–59.9	19	50.5 ± 14.9	39.9–61.2	10	55.1 ± 15.6	43.1–67.1	10
Blood gas analysis										
SaO_2_ (%)		92.9 ± 3.5	91.3–94.6	19	91.8 ± 4.3	88.5–95.0	10	94.01 ± 2.36	92.32–95.7	10
PaO_2_ (%)		66.0 ± 12.3	60.3–71.7	20	62.3 ± 9.6	55.4–69.2	10	69.7 ± 13.9	59.8–79.6	10
PaCO_2_ (%)		34.6 ± 6.2	31.7–37.5	20	36.3 ± 6.7	31.5–41.1	10	32.97 ± 5.39	29.11–36.83	10
Laboratory										
Hemoglobin (g/dl)		14.1 ± 2.0	13.1–15.1	18	14.3 ± 1.9	12.9–15.8	10	13.8 ± 2.2	12.1–15.5	10
Hematocrit (1/L)		0.4 ± 0.1	0.4–0.4	18	0.4 ± 0.1	0.4–0.5	10	0.4 ± 0.1	0.4–0.5	10
Platelets (/nl)		197.2 ± 85.6	154.6–239.7	18	185.2 ± 47.6	148.6–221.8	9	209.1 ± 113.9	121.5–296.7	9
Creatinine (mg/dl)		1.1 ± 0.4	0.9–1.3	18	1.2 ± 0.4	0.9–1.5	9	1.1 ± 0.3	0.8–1.3	9
Glomerular filtration rate (ml/min/1.73 m^2^)		65.2 ± 20.8	54.9–75.6	18	60.7 ± 26.5	40.4–81.1	9	69.7 ± 13.31	59.5–79.9	9
C-reactive protein (mg/l)		6.9 ± 6.1	3.8–10.1	17	8.6 ± 8.5	1.5–15.7	8	5.5 ± 2.7	3.4–7.5	9
N-terminal pro brain natriuretic peptide (ng/l)		1388.4 ± 1974.9	372.9–2403.8	17	1803.8 ± 2273.1	56.5–3551.0	9	921 ± 1593.2	-410.9–2252.9	8
6-minute walking distance (meters)		370.4 ± 98.1	321.6–419.2	18	387.9 ± 75.0	325.2–450.6	8	356.4 ± 115.3	273.9–438.9	10
Borg Dyspnea-Scale (out of 10)		3.9 ± 2.1	2.9–4.9	18	3.3 ± 1.5	2.1–4.6	8	4.4 ± 2.4	2.6–6.1	10

*In case of missing values, n is provided.

*CO* carbon monoxide, *CO*_*2*_ carbon dioxide, *O*_*2*_ oxygen, *Pa* partial pressure, *Sa* saturation, *SB* single breath, *SD* standard deviation, *VA* alveolar volume, *WHO FC* World Health Organization functional class.

**Table 2:** Further clinical parameter of the study cohort at baseline.

	Whole cohort (n = 20)			Oxygen treatment (n = 10)			Control group (n = 10)		
	Mean ± SD or n and (%)	95% Confidence interval	n*	Mean ± SD or n and (%)	95% Confidence interval	n*	Mean ± SD or n and (%)	95% Confidence interval	n*
Echocardiography									
Right atrial area (cm^2^)	15.9 ± 4.5	13.8–18.0	20	15.9 ± 3.3	13.6–18.2	10	15.9 ± 5.6	11.9–19.9	10
Right ventricular area (cm^2^)	18.9 ± 5.3	16.4–21.4	20	18.8 ± 3.3	16.4–21.2	10	19.0 ± 7.0	14.0–24.0	10
Systolic pulmonary arterial pressure (mmHg)	52.7 ± 14.0	46.2–59.2	20	52.4 ± 12.0	43.8–61.0	10	53.0 ± 16.4	41.3–64.7	10
Tricuspid annular plane systolic excursion (cm)	2.18 ± 0.4	2.0–2.35	20	2.27 ± 0.3	2.08–2.5	10	2.08 ± 0.4	1.8–2.4	10
Left ventricular eccentricity index (LV-EI)	1.11 ± 0.1	1.04–1.18	18	1.13 ± 0.1	1.03–1.2	10	1.1 ± 0.2	1.0–1.2	8
Right heart catheter									
Mean pulmonary arterial pressure (mmHg)	39.7 ± 12.5	33.7–45.7	19	38.0 ± 11.0	30.1–45.9	10	41.6 ± 14.4	30.5–52.6	9
Cardiac output (L/min)	5.4 ± 1.3	4.77–6.03	19	5.62 ± 1.4	4.6–6.64	10	5.2 ± 1.2	4.2–6.1	9
Pulmonary arterial wedge pressure (mmHg)	9.4 ± 3.7	7.6–11.2	19	9.7 ± 4.8	6.2–13.2	10	9.0 ± 2.1	7.4–10.6	9
Pulmonary vascular resistance (WU)	507.4 ± 327.5	349.5–665.2	19	422.1 ± 190.9	285.5–558.7	10	602.1 ± 425.7	274.9–929.3	9
Cardiac index (l/min/m^2^)	2.8 ± 0.6	2.5–3.1	19	2.8 ± 0.7	2.3–3.3	10	2.7 ± 0.6	2.3–3.2	9
Right ventricular pump function									
Normal	11 ± 55%								
Mild impairment	2 ± 10%								
Moderate impairment	2 ± 10%								
Severe impairment	5 ± 25%								
Quality of life (SF-36)									
Physical functioning	49.5 ± 29.7	35.2–63.8	19	53.9 ± 18.7	39.5–68.2	9	45.5 ± 37.6	18.6–72.4	10
Physical role function	50 ± 42.5	29.5–70.5	19	55.6 ± 34.9	28.8–82.4	9	45.0 ± 49.7	9.4–80.6	10
Pain	75.5 ± 33.6	59.3–91.7	19	82 ± 31.7	57.6–106.4	9	69.6 ± 35.9	44–95.2	10
General health perception	45.2 ± 18.7	36.2–54.2	19	51.6 ± 19.3	36.7–66.4	9	39.5 ± 17.1	27.2–51.8	10
Vitality	48.9 ± 18.8	39.9–58	19	52.8 ± 13.0	42.8–62.8	9	45.5 ± 22.9	29.1–61.9	10
Social functioning	71.2 ± 25.6	58.9–83.5	19	82.1 ± 24.9	62.9–101.3	9	61.4 ± 23.1	44.9–77.9	10
Emotional role function	52.6 ± 47.6	29.6–75.5	19	66.6 ± 40.9	35.1–98	9	40.0 ± 51.6	3.1–76.9	10
Mental well-being	62.5 ± 20.6	52.6–72.5	19	64.4 ± 14.9	53–75.9	9	60.8 ± 25.4	42.6–79	10
Physical summation score	53.3 ± 22.7	42.4–64.3	19	58.9 ± 14.6	47.7–70.1	9	48.3 ± 28.0	28.3–68.3	10
Mental summation score	56 ± 22.3	45.3–66.7	19	63.7 ± 17.5	50.2–77.1	9	49.1 ± 24.7	31.4–66.8	10

*In case of missing values, n is provided.

*SD* standard deviation.

A total of 31 patients were pre-screened and 22 patients initially met the inclusion criteria; one patient dropped out before randomization and one patient was a screening failure. Consequently 20 patients, 10 in each group were randomized (flow-chart, Fig. 1), 14 (70%) women, 12 idiopathic PAH, 3 CTEPH (2 inoperable, 1 recurrent), 4 associated PAH with connective tissue diseases, 1 portopulmonary hypertension (Table 1). Clinical parameters at baseline are summarized in Tables 1 and 2.

One patient of the control group received newly initiated PAH targeted treatment at baseline, which may have led to an improvement of clinical parameters during the control period. One further patient of the intervention group significantly reduced O_2_ treatment after baseline due to an adverse event (headache). Both patients were considered in the intention-to-treat, but not in the per-protocol analysis set.

### O_2_ flow rate and adherence

One patient of the intervention group stopped O_2_ treatment one day after the final study assessment, during safety follow-up. One further patient reduced O_2_ flow after baseline. One patient died 92 days after beginning of O_2_ treatment. All other patients continued O_2_ treatment even after the final study assessment. Seven patients started LTOT as secondary intervention group.

### Primary outcome

**Fig. 2:**
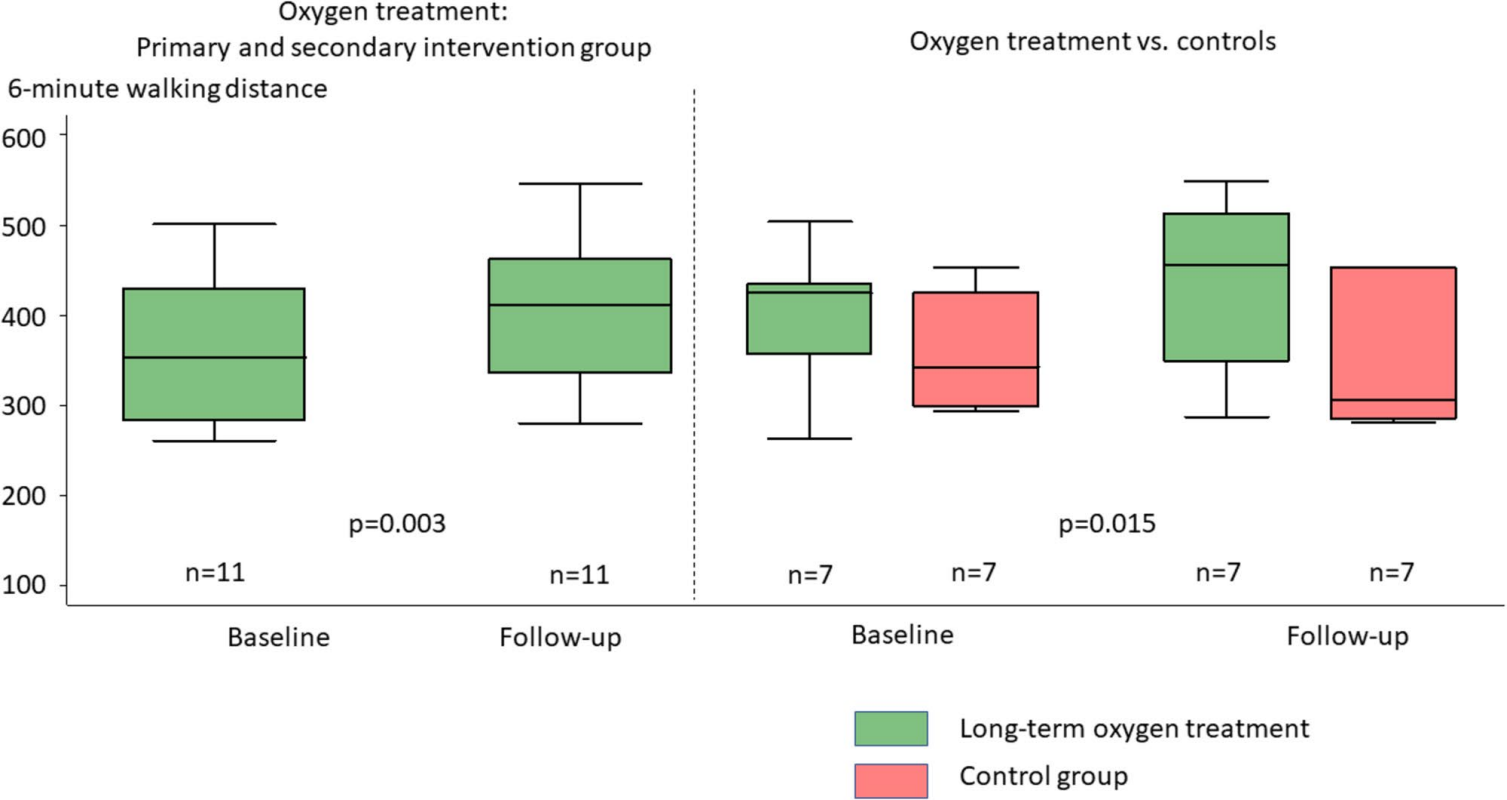
Boxplots on the primary endpoint 6-min walking distance. Patients significantly improved 6-minute walking distance under long-term oxygen treatment for both intra-individual changes of primary and secondary intervention group, as well as for oxygen treatment vs. controls.

The 6MWD significantly improved in patients receiving O_2_ treatment both in the intra-individual comparison of all patients (42.2 ± 34.2 m, *p* = 0.003, Table 3), and in the intervention vs. control group (O_2_ 38.9 ± 33.87 m vs. control − 12.3 ± 21.83 m, *p* = 0.015, Fig. 2, Table 4). The per protocol analysis confirmed the results (*p* = 0.003 LTOT in primary and secondary intervention group; *p* = 0.015 LTOT vs. no O_2_). Analysis of the primary endpoint 6MWD with O_2_ administration showed a trend in improvement for all patients receiving LTOT (all patients: 35 ± 81.6 m improvement by LTOT; *p* = 0.091) and for the intervention vs. control group (O_2_ group 47.8 ± 88.9 m vs. control − 11.5 ± 21.4 m).

**Table 3:** Changes during long term oxygen treatment (pooled sample, primary and secondary intervention group).

		Long-term oxygen treatment (n = 14)		
		Mean ± SD or n and (%)	95% Confidence interval	n*
Lung function				
Vital capacity max (l)		− 0.032 ± 0.372	− 0.247 to 0.182	
Forced expiratory volume in one second (l)		− 0.040 ± 0.187	− 0.148 to 0.068	
Total lung capacity (l)		0.235 ± 0.847	− 0.254 to 0.724	
Diffusion capacity of the lung for CO SB (mmol/min/kPa)		− 1.575 ± 7.04	− 5.64 to 2.49	
Diffusion capacity of the lung for CO/VA (%)		2.64 ± 10.14	− 3.21 to 8.50	
Blood gas analysis				
SaO_2_ (%)		− 0.02 ± 3.32	− 2.02 to 1.99	13
PaO_2_ (%)		1.9 ± 7.04	− 2.2 to 5.9	
PaCO_2_ (%)		1.18 ± 2.53	− 0.28 to 2.64	
Laboratory				
Hemoglobin (g/dl)		− 0.99 ± 2.58	− 2.55 to 0.57	13
Hematocrit (1/L)		0.002 ± 0.048	− 0.026 to 0.031	13
Platelets (/nl)		− 1.2 ± 39.23	− 24.9 to 22.5	13
Creatinine (mg/dl)		0.038 ± 0.197	− 0.081 to 0.157	13
Glomerular filtration rate (ml/min/1.73 m^2^)		1.30 ± 9.33	− 4.34 to 6.95	13
C-reactive protein (mg/l)		− 1.33 ± 4.24	− 4.02 to 1.37	12
N-terminal pro brain natriuretic peptide (ng/l)		470.2 ± 1515.5	− 445.6 to 1386	13
6-minute walking distance (meters)		42.2 ± 34.2	19.2 to 65.2^#^	11
Borg Dyspnea-Scale (out of 10)		0.4 ± 1.56	− 0.6 to 1.5	11
Echocardiography				
Right atrial area (cm^2^)		− 0.4 ± 2.92	− 2.0 to 1.3	
Right ventricular area (cm^2^)		− 0.9 ± 4.05	− 3.3 to 1.4	
Systolic pulmonary arterial pressure (mmHg)		− 5.3 ± 16.58	− 14.9 to 4.3	
Tricuspid annular plane systolic excursion (mm)		1.4 ± 5.66	− 1.8 to 4.7	
Left ventricular eccentricity index (LV-EI)		0 ± 0.17	− 0.1 to 0.1	
Right heart catheter				
Mean pulmonary arterial pressure (mmHg)		− 1.86 ± 9.4	− 7.28 to 3.57	
Cardiac output (L/min)		− 0.15 ± 0.93	− 0.68 to 0.39	
Pulmonary arterial wedge pressure (mmHg)		0.86 ± 3.28	− 1.04 to 2.75	
Pulmonary vascular resistance (dyn × sec × cm^−5^)		− 38.9 ± 108.95	− 101.8 to 24.1	
Cardiac index (l/min/m^2^)		0.04 ± 0.55	− 0.28 to 0.35	
Right ventricular pump function				
Normal		− 1 ± − 7.1		
Mild impairment		1 ± 7.1		
Moderate impairment		0 ± 0		
Severe impairment		0 ± 0		
Quality of life (SF-36)				
Physical functioning		15.0 ± 28.21	− 2.0 to 32.0	13
Physical role function		17.3 ± 41.31	− 7.7 to 42.3	13
Pain		− 5.2 ± 15.51	− 14.6 to 4.1	13
General health perception		7.9 ± 22.60	− 5.7 to 21.6	13
Vitality		4.6 ± 22.03	− 8.7 to 17.9	13
Social functioning		− 1.9 ± 23.72	− 16.3 to 12.4	13
Emotional role function		12.9 ± 39.78	− 11.1 to 37.0	13
Mental well-being		13.8 ± 22.19	0.4 to 27.3^#^	13
Physical summation score		8.0 ± 19.04	− 3.5 to 19.5	13
Mental summation score		7.5 ± 19.41	− 4.2 to 19.3	13

*In case of missing values, n is provided, ^#^Confidence interval does not include “0”.

*CO* carbon monoxide, *CO*_*2*_ carbon dioxide, *O*_*2*_ oxygen, *Pa* partial pressure, *Sa* saturation, *SB* single breath, *SD* standard deviation, *VA* alveolar volume, *WHO FC* World Health Organization functional class.

**Table 4:** Treatment effects of long-term oxygen treatment (changes in intervention and control group).

		Oxygen treatment (n = 10)	Control group (n = 10)	*p* value			
		Mean ± SD or n and (%)	95% Confidence interval	n*	Mean ± SD or n and (%)	95% Confidence interval	n*
Lung function							
Vital capacity max (l)	− 0.118 ± 0.307	− 0.354 to 0.118	9	− 0.071 ± 0.409	− 0.450 to 0.307	7	0.459
Forced expiratory volume in one second (l)	− 0.076 ± 0.188	− 0.220 to 0.069	9	− 0.106 ± 0–317	− 0.398 to 0.187	7	0.832
Total lung capacity (l)	0.248 ± 1.073	− 0.577 to 1.073	9	− 0.201 ± 0.503	− 0.666 to 0.263	7	0.525
Diffusion capacity of the lung for CO SB (mmol/min/kPa)	0.16 ± 0.61	− 0.31 to 0.63	9	− 0.51 ± 15.63	− 14.97 to 13.94	7	0.832
Diffusion capacity of the lung for CO/VA (%)	1.73 ± 7.89	− 4.33 to 7.80	9	− 0.83 ± 11.94	− 11.87 to 10.22	7	0.916
Blood gas analysis							
SaO_2_ (%)	− 0.28 ± 3.96	− 3.58 to 3.03	8	0.66 ± 2.02	− 1.02 to 2.35	8	0.958
PaO_2_ (%)	2.8 ± 6.02	− 1.8 to 7.4	9	− 2.3 ± 13.03	− 13.2 to 8.6	8	0.335
PaCO_2_ (%)	0.21 ± 2.02	− 1.34 to 1.77	9	− 1.53 ± 2.46	− 3.58 to 0.53	8	0.268
Laboratory							
Hemoglobin (g/dl)	− 0.40 ± 1.42	− 1.58 to 0.78	8	− 0.41 ± 1.11	− 1.44 to 0.61	7	0.862
Hematocrit (1/L)	− 0.005 ± 0.041	− 0.039 to 0.029	8	− 0.003 ± 0.036	− 0.037 to 0.031	7	0.723
Platelets (/nl)	9.6 ± 28.92	− 14.6 to 33.8	8	47.1 ± 42.96	7.4 to 86.9	7	0.118
Creatinine (mg/dl)	0.03 ± 0.21	− 0.14 to 0.21	8	− 0.05 ± 0.16	− 0.20 to 0.10	7	0.953
Glomerular filtration rate (ml/min/1.73 m^2^)	3.17 ± 8.06	− 3.57 to 9.91	8	3.95 ± 8.54	− 3.94 to 11.85	7	0.908
C-reactive protein (mg/l)	− 2.70 ± 4.81	− 7.15 to 1.75	7	− 1.43 ± 2.57	− 3.81 to 0.95	7	1.00
N-terminal pro brain natriuretic peptide (ng/l)	459.8 ± 1798.0	− 1043.4 to 1962.9	8	− 313.0 ± 857.0	− 1105.6 to 479.6	7	0.603
6-minute walking distance (meters)	38.9 ± 33.87	7.5 to 70.2	7	− 12.3 ± 21.83	− 32.5 to 7.9	7	0.015
Borg Dyspnea-Scale (out of 10)	0.2 ± 1.22	− 0.9 to 1.3	7	0.5 ± 1.19	− 0.6 to 1.6	7	0.946
Echocardiography							
Right atrial area (cm^2^)	0.3 ± 3.32	− 2.2 to 2.9	9	1.6 ± 1.77	0.1 to 3.1	8	0.2
Right ventricular area (cm^2^)	− 0.7 ± 4.09	− 3.8 to 2.5	9	0.5 ± 2.14	− 1.3 to 2.3	8	0.12
Systolic pulmonary arterial pressure (mmHg)	− 2.7 ± 15.01	− 14.2 to 8.9	9	6.9 ± 19.63	− 9.5 to 23.3	8	0.282
Tricuspid annular plane systolic excursion (cm)	2.32 ± 7.03	− 3.08 to 7.72	9	0.46 ± 0.41	0.12 to 0.81	8	0.176
Left ventricular eccentricity index (LV-EI)	− 0.02 ± 0.21	− 0.18 to 0.14	9	0.10 ± 0.20	− 0.08 to 0.28	7	0.32
Right heart catheter							
Mean pulmonary arterial pressure (mmHg)	− 1.78 ± 11.25	− 10.42 to 6.87	9	− 2.57 ± 8.22	− 10.18 to 5.03	7	0.363
Cardiac output (L/min)	64.43 ± 193.94	− 86.64 to 213.51	9	8.07 ± 21.00	− 11.36 to 27.50	7	0.289
Pulmonary arterial wedge pressure (mmHg)	0.78 ± 2.86	− 1.42 to 2.98	9	− 0.14 ± 2.34	− 2.31 to 2.02	7	0.665
Pulmonary vascular resistance (dynes*sec*cm^−5^)	− 47.0 ± 133.95	− 150.0 to 56.0	9	− 100.7 ± 253.17	− 334.9 to 133.4	7	0.832
Cardiac index (l/min/m^2^)	42.16 ± 126.39	− 55.0 to 139.31	9	0.09 ± 0.241	− 0.14 to 0.31	7	1.00
Quality of life (SF-36)							
Physical functioning	21.9 ± 31.84	− 4.7 to 48.5	8	− 5.0 ± 7.64	− 12.1 to 2.1	7	0.091
Physical role function	15.6 ± 42.13	− 19.6 to 50.8	8	− 17.9 ± 31.34	− 46.8 to 11.1	7	0.116
Pain	− 6.4 ± 16.07	− 19.8 to 7.1	8	− 6.0 ± 30.09	− 33.8 to 21.8	7	0.900
General health perception	9.1 ± 28.74	− 14.9 to 33.2	8	− 4.7 ± 14.63	− 18.2 to 8.8	7	0.383
Vitality	0.6 ± 24.27	− 19.7 to 20.9	8	− 5.7 ± 13.97	− 18.6 to 7.2	7	0.522
Social functioning	− 3.3 ± 25.50	− 24.6 to 18.1	8	3.4 ± 15.58	− 11.0 to 17.8	7	0.540
Emotional role function	8.5 ± 38.87	− 24.0 to 41.0	8	14.3 ± 37.80	− 20.7 to 49.2	7	0.629
Mental well-being	14.5 ± 23.12	− 4.8 to 33.8	8	− 4.0 ± 9.80	− 13.1 to 5.1	7	0.180
Physical summation score	8.3 ± 20.60	− 9.0 to 25.5	8	− 7.9 ± 13.73	− 20.6 to 4.8	7	0.118
Mental summation score	5.8 ± 21.35	− 12.1 to 23.6	8	0.6 ± 13.70	− 12.1 to 13.2	7	0.684

*In case of missing values, n is provided.

*CO* carbon monoxide, *CO*_*2*_ carbon dioxide, *O*_*2*_ oxygen, *Pa* partial pressure, *Sa* saturation, *SB* single breath, *SD* standard deviation, *VA* alveolar volume, *WHO FC* World Health Organization functional class.

### Secondary outcomes (Tables 3 and 4)

The FEV1/maximal vital capacity (FEV1/% VC max) showed a trend of improvement with an increase in the intervention group and a decrease in the control group (*p* = 0.09). This result was, however, not confirmed by analysis of the pooled data of patients receiving LTOT, with no significant change in lung function parameters (Tables 3 and 4).

In the per protocol analysis, the intervention group showed a significant decrease in sPAP (*p* = 0.020) and mPAP (*p* = 0.019) compared to the control group and a trend in decrease of heart rate (*p* = 0.055, data not shown). This finding was, however, not confirmed with the intention-to-treat analysis set. Furthermore, patients receiving O_2_ had a trend of improvement in physical functioning (SF-36 questionnaire, *p* = 0.091, Table 4), which was more pronounced in the per protocol analysis (*p* = 0.07, data not shown), but could not be detected in the intra-individual effect of LTOT. In the per protocol analysis, there was also a trend of improvement in physical role function (*p* = 0.067) and physical summation score (*p* = 0.073, data not shown).

### Adverse events and tolerability (Table 5)

**Table 5:** Adverse events.

Adverse events (if ≥ 10% of patients)	Oxygen treatment (primary and secondary intervention group)	Control group
Serious adverse events		
SARS-CoV_2_ infection	1 (fatal due to pneumonia)	–
*Staph. aureus* bacteremia	1	–
Hospitalisation due to renal insufficiency	1	–
Anemia	1	–
Adverse events		
SARS-CoV_2_ infection	3 (1 SAE)	–
Epistaxis	2	–
Headache	1*	1
Vaccination reaction after SARS-CoV_2_-vaccination	2	2

*Possible association with O_2_ Therapy.

LTOT was generally well tolerated. In the intervention group, 12 adverse events were reported, while 4 events occurred in the control group. The most frequent adverse event was a reaction to SARS-CoV_2_ vaccination (Table 5). One patient reported intermittent, mild headache under LTOT. Further adverse events were reported to be not associated with study medication. One patient from the cross-over O_2_ group developed a bacteremia and subsequent anemia. Two patients died during the study, one patient due to SARS-CoV_2_ pneumonia in the O_2_ arm and one due to renal insufficiency that led to right heart failure in the control group (Table 5). All these severe adverse events were reported as not associated with the study medication.

## Discussion

The SOPHA study was the first prospective, randomized, controlled, parallel-group clinical trial assessing the effect of LTOT over 12 weeks in patients with PAH and recurrent or persistent CTEPH with mild hypoxemia at rest and/or during exercise including hemodynamic measurements under targeted PH therapy. LTOT led to a significant and clinically relevant improvement of exercise capacity of 42 m within 3 months after initiation of LTOT. Under O_2_ substitution there were no significant changes in functional class, hemodynamics, echocardiographic and laboratory parameters. A tendency for improvement of QoL could be shown, in particular of physical functioning scores. Compared to patients who did not receive LTOT, patients receiving LTOT profited, with a significant improvement of 6MWD of > 40 m over the study period.

Hypoxemia in PAH is usually associated with ventilation-perfusion mismatch, reduced mixed venous oxygen saturation, reduced diffusion capacity, shunts, congenital heart failure and hepatic diseases^14^. Robust data for LTOT in PAH and CTEPH are lacking^1^. O_2_ therapy received a class I level C recommendation in the current guidelines, in case the arterial partial O_2_ pressure at rest is < 60 mmHg, based on evidence from previous COPD studies^1^. Patients with PAH/CTEPH and hypoxemia during exercise benefit in terms of exercise capacity shown in 6MWD, quality of life and functional class, from standardized, low-flow O_2_ substitution given at rest^8^. On the other hand, most patients with Eisenmenger syndrome did not profit from O_2_ supply, most probably explained by the intracardiac shunt which did not allow to increase SpO_2_^5^. However, the effects of O_2_ therapy remain contradictory.

As oxygen acts as a potent pulmonary vasodilator^14^, further treatment benefits could lie in an improvement of muscle economics and strength by increased oxygenation.

In the present study, LTOT led to significant improvement of 6MWD independently of O_2_ substitution during the exercise test. Significant improvement could be seen for both all patients receiving O_2_ (intra-individual comparison) and the intervention vs. control group for 6MWD without and with O_2_. Intra-individual comparison with O_2_ administration during the 6MWD showed an improvement in trend, which may have been caused by the small sample size or the additive difficulty of carrying or pulling an oxygen device during the test. Compared to previous studies among PH patients, FC did not improve over the course of the study. There was only a tendency in terms of improvement of physical functioning scales in the QoL questionnaire. The results of this study assist the long-term O_2_ substitution in patients with PAH and mild hypoxemia at rest and/or during exercise. An improvement of standard of care through optimisation of O_2_ substitution through health insurance providers is warranted.

In this study, only patients with mild hypoxia at rest and during exercise were included as including patients with severe desaturation in a randomized, controlled trial with the chance of a 12-week delay of O_2_ supplementation within the control group would seem unethical. This may have influenced the effect of LTOT, as hypoxia was only mild.

In our study two patients died, one due to severe SARS-CoV_2_ associated pneumonia in the LTOT arm and one due to renal insufficiency that led to right heart failure in the control group. Previous studies^2,15^ showed a significant improvement of hemodynamics in particular of PVR after acute exposure with O_2_ in patients with PAH/CTEPH. In this study, the assessments during RHC were performed without O_2_ in order to measure the long-term effect of O_2_ and not the acute effect, which has been described previously^15,16^. There was no significant change in the hemodynamic parameters in the intention to treat group, although there was a significant improvement of mPAP (*p* = 0.020), sPAP (*p* = 0.019) and improvement of heart rate (*p* = 0.055) in the intervention group in the per protocol assessment.

Furthermore, no significant changes in echocardiographic, WHO FC, PFT and laboratory parameters (i.e. NTproBNP) were observed. Quality of life data indicate a potential benefit regarding physical function related quality of life scales. Effects of LTOT on clinical parameters should be conducted in future research with larger-scaled randomized controlled trials.

The strengths of this study include the randomized, controlled study design with blinding of assessors of the primary endpoint. The performance of a monocentric clinical trial demonstrated several limitations. As mild hypoxemia is rarely seen among patients with PAH and CTEPH, recruitment difficulties were present. Furthermore, the study was performed during the SARS-CoV_2_ pandemic, resulting in a recruitment stop over a longer time-period and leading to an amendment with reduction of recruited patients and statistical power, as the trial was performed in a single centre. Furthermore, CPET for the evaluation of O_2_ uptake was removed from the study assessments in these patients, which would have been desirable for interpretation of LTOT effects. The patients were not blinded during the trial, which may have led to biased results and early withdrawal in the no O_2_ arm (Fig. 1). Furthermore, efficacy data was not present from all patients, which may have been caused by duplicate assessments in the case of the 6MWD with and without O_2_ supply. In addition, the study was not powered to detect significant changes in other clinical parameters. Due to the small sample size, the effect of LTOT was analysed in all patients with precapillary PH without differentiation of subtypes, which does not allow conclusions on different effect sizes. Therefore, the clinical effect of LTOT should be assessed in larger clinical trials. Further studies are needed to distinguish the effect in different diagnostic subgroups and to further elaborate modes of action in these patients. Due to the small sample size, the effect of LTOT was analysed in all patients with precapillary PH without differentiation of subtypes, which does not allow conclusions on different effect sizes.

This study highlights the importance of LTOT in terms of exercise capacity in patients with PAH and CTEPH. A statistically significant and clinically relevant improvement of 6MWD was shown independent from the acute substitution in both all patients receiving LTOT and in the LTOT vs. control group. Effects of LTOT on QoL, hemodynamics, echocardiographic and laboratory parameters should be investigated in further, larger-scaled trials.

## Data Availability

The datasets used and/or analysed during the current study are available upon reasonable request to the corresponding author.

## Abbreviations

6MWD: 6-Min walking distance
BfARM: Bundesinstitut für Arzneimittel und Medizinprodukte
BGA: Blood gas analysis
CI: Cardiac index
CO: Cardiac output
CPET: Cardiopulmonary exercise test
CTEPH: Chronic thrombo-embolic pulmonary hypertension
DLCO: Diffusion capacity of carbon monoxide
dPAP: Diastolic pulmonary arterial pressure
ERS: European Respiratory Society
ESC: European Society of Cardiology
FEV_1_: Forced expiratory volume in the first second
LTOT: Long-term oxygen therapy
LV-EI: Left-ventricular eccentricity index
mPAP: Mean pulmonary arterial pressure
NTproBNP: N-terminal pro-brain natriuretic peptide
PAH: Pulmonary arterial hypertension
PAWP: Pulmonary arterial wedge pressure
PFT: Pulmonary function test
PH: Pulmonary hypertension
pO_2_: Partial pressure of oxygen
PVR: Pulmonary vascular resistance
QoL: Quality of life
RA: Right atrium
RAP: Right atrial pressure
RHC: Right heart catheterisation
RV: Right ventricle
SD: Standard deviation
SF-36: Short form 36 health survey
sPAP: Systolic pulmonary arterial pressure
SpO_2_: Saturation of peripheral oxygen
SvO_2_: Central venous oxygen saturation
TAPSE: Tricuspid annular plane systolic excursion
TLC: Total lung capacity
VCmax: Maximal vital capacity
WHO-FC: World Health Organization functional class
WU: Wood unit(s)
